# The coupling between healthspan and lifespan in *Caenorhabditis* depends on complex interactions between compound intervention and genetic background

**DOI:** 10.18632/aging.205743

**Published:** 2024-04-12

**Authors:** Stephen A. Banse, E. Grace Jackson, Christine A. Sedore, Brian Onken, David Hall, Anna Coleman-Hulbert, Phu Huynh, Theo Garrett, Erik Johnson, Girish Harinath, Delaney Inman, Suzhen Guo, Mackenzie Morshead, Jian Xue, Ron Falkowski, Esteban Chen, Christopher Herrera, Allie J. Kirsch, Viviana I. Perez, Max Guo, Gordon J. Lithgow, Monica Driscoll, Patrick C. Phillips

**Affiliations:** 1 Institute of Ecology and Evolution, University of Oregon, Eugene, OR 97403, USA; 2 Department of Molecular Biology and Biochemistry, Rutgers, The State University of New Jersey, Piscataway, NJ 08854, USA; 3 The Buck Institute for Research on Aging, Novato, CA 94945, USA; 4 Division of Aging Biology, National Institute on Aging, Bethesda, MD 20892, USA

**Keywords:** aging, healthspan, genetic diversity, compound intervention

## Abstract

Aging is characterized by declining health that results in decreased cellular resilience and neuromuscular function. The relationship between lifespan and health, and the influence of genetic background on that relationship, has important implications in the development of pharmacological anti-aging interventions. Here we assessed swimming performance as well as survival under thermal and oxidative stress across a nematode genetic diversity test panel to evaluate health effects for three compounds previously studied in the *Caenorhabditis* Intervention Testing Program and thought to promote longevity in different ways – NP1 (nitrophenyl piperazine-containing compound 1), propyl gallate, and resveratrol. Overall, we find the relationships among median lifespan, oxidative stress resistance, thermotolerance, and mobility vigor to be complex. We show that oxidative stress resistance and thermotolerance vary with compound intervention, genetic background, and age. The effects of tested compounds on swimming locomotion, in contrast, are largely species-specific. In this study, thermotolerance, but not oxidative stress or swimming ability, correlates with lifespan. Notably, some compounds exert strong impact on some health measures without an equally strong impact on lifespan. Our results demonstrate the importance of assessing health and lifespan across genetic backgrounds in the effort to identify reproducible anti-aging interventions, with data underscoring how personalized treatments might be required to optimize health benefits.

## INTRODUCTION

Age-associated health deterioration results in increased prevalence of numerous diseases [1], and reduced muscle function [2], movement [3], and stress resistance [4]. Aging is also a principal risk factor for mortality, with hazard rates increasing throughout adulthood. Retarding the fundamental biology of aging has been suggested as a remedy to improve health in old age [5]. However, how health (often measured by a range of metrics) relates to longevity remains surprisingly unclear; how pharmacological interventions perturb this relationship adds another layer of complexity, and how genetic diversity interfaces with chemical interventions aimed at health and longevity introduces an additional dimension that complicates understanding in both the basic biology and anti-aging treatment arenas.

One challenge in dissecting the relationships among longevity, health, and genetic background is the difficulty in defining health in model systems. For human health, integrative approaches using physical, cognitive, or physiological performance are used as a proxy for health state. These approaches include the Short Physical Performance Battery (an assessment of gait speed, chair stand, and balance in the elderly [6–8]), Frailty Index (assessed by presence of disease, physical disability, and cognitive decline [9, 10]), and the Healthy Aging [11], Successful Aging [12], and Cognitive Frailty indices [13]. Parallel approaches have been suggested for invertebrate models (e.g., *Drosophila* locomotion [14] vs. human treadmill testing) in which genetics can be controlled, thus facilitating study of both the relationship between health and lifespan, and the influence of genetic background.

*Caenorhabditis elegans* is a widely used research model that experiences age-dependent declines in a variety of physiological processes [15, 16]. A number of health assays have been employed in *C. elegans* to quantify functional declines. Among the most commonly used health-related phenotypes are stress resistance and body movement [15–22] (measures that broadly align with health and intrinsic capacity [13, 23]), pharyngeal pumping [24, 25], and accumulation of autofluorescent granules [22, 26]. These measures have been used to probe the relationship between health and lifespan [20, 27, 28]. For example, measures of physiological function like thermotolerance and oxidative stress resistance can positively correlate with longevity [20, 29, 30]. Motility, a measure of neuromuscular function, is positively correlated with lifespan in some, but not all, studies [3, 16, 17].

How, and if, lifespan and health measures are related thus remains hotly debated. There are a number of definitions of healthspan, with healthspan generally defined as the length of time before a precipitous loss in stress resistance and physiological function. Previous studies have shown that compound interventions that improve healthspan do not necessarily impact lifespan [31–33]. Recent work that used a definition of healthspan based on population maximum lifespans suggests that in a number of long-lived mutants, including well-characterized IIS/IGF signaling pathway mutants, healthspan can be largely uncoupled from lifespan [18]. Subsequent work and revisited analyses have contradicted these results, finding that healthspan, at least in long-lived *daf-2* mutants, is maintained proportionally with lifespan, even as other long-lived mutants exhibit attenuated health outcomes [17].

On top of these complications, the influence of genetic background remains a relatively unexplored variable in aging biology because studies have largely been constrained to a single isogenic line, namely *C. elegans* laboratory strain N2. However, genetic background plays a critical role in determining the effects of different pharmacological interventions on lifespan [34], and compounds may affect health in species- and even population-specific ways. The *Caenorhabditis* Intervention Testing Program (CITP) is anchored in a collaborative approach to address the complexities inherent in testing pharmaceutical impact on longevity and health across genetic backgrounds by exploiting the diversity of the *Caenorhabditis* genus. Using representatives of three *Caenorhabditis* species that encompass genetic variation similar to that between mice and humans [35–37], the CITP implements identical protocols at three independent sites to screen for small molecule lifespan and healthspan effects, with the goal of minimizing lab-to-lab variability to generate high quality, reproducible results. CITP published data demonstrate the efficacy of this approach: in an initial screen of 12 compounds for longevity effects, we identified 6 that reproducibly promote lifespan in at least one of the tested species [34].

Here we set out to apply CITP protocols to answer three questions for compounds with potential anti-aging effects: (1) When a compound extends lifespan, does it do so by fundamentally slowing the aging process, resulting in broad health benefits? (2) Do compound interventions promote health benefits in genetic backgrounds that do not exhibit lifespan extension? and (3) What is the relationship between health measures and lifespan, and which health measures are most reproducible and informative for future CITP compound evaluation? To answer these questions, we determined health effects for three pro-longevity compounds tested in previous CITP screens [34, 38]: candidate dietary restriction mimetic NP1, oxidative stress pathway-implicated propyl gallate, and red wine component resveratrol. We assessed health by measuring survival under heat stress, survival under oxidative stress, and swimming performance. We find that swimming ability and oxidative stress resistance are highly reproducible across labs, but that compound intervention effects on oxidative stress and swimming ability do not correlate with lifespan effects across genetic backgrounds. In contrast, compound interventions do intersect with thermotolerance in a manner that correlates with longevity. Our results demonstrate the value of assessing health declines across genetically diverse test sets in the search for reproducible anti-aging interventions.

## RESULTS

**Figure 1:**
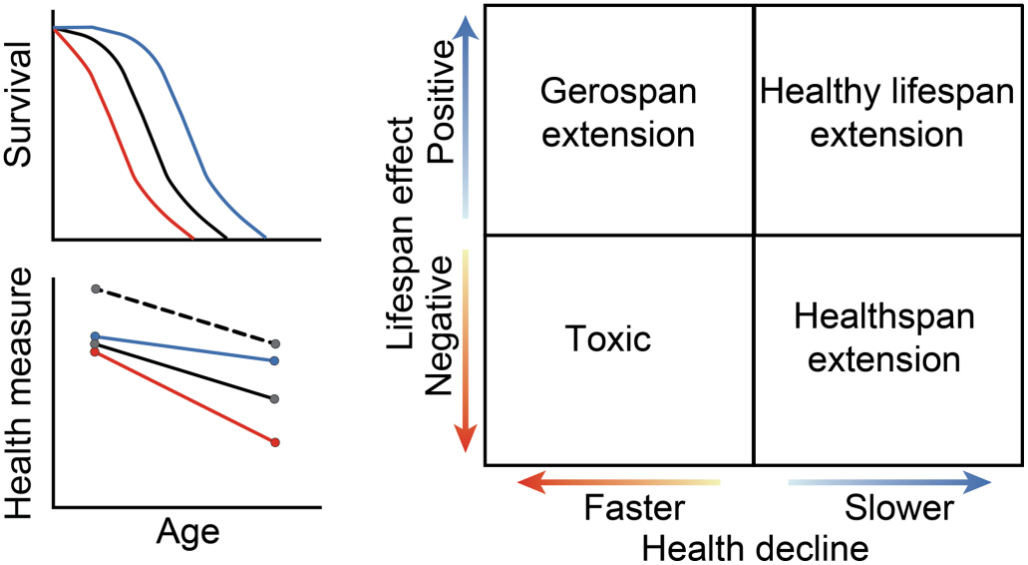
**Potential effects of compounds on lifespan and health.** A qualitative diagram of possible outcomes for compound effects on lifespan and on healthspan. Lifespan is represented as a change in median survival, while healthspan is represented in the relative rate of decline as compared to the control. The black lines show control, while the blue lines depict slowed aging, and the red lines depict accelerated aging. For health measures, the black dashed line shows the effects of an intervention that is generally stimulatory but does not alter the aging process. Depending on the effect size and direction, each healthspan, compound, and strain combination will fall into one quadrant: lifespan and healthspan extending, healthspan extending, gerospan extending, or toxic. The solid lines between the quadrants indicate no change from the control for a given measure.

Aging is marked by a progressive decline in physiological function and an increase in hazard rate [39]. An anti-aging intervention treating the root cause(s) of aging would slow those progressive changes, resulting in larger relative improvements late in life, rather than simply being stimulatory at all ages. Anti-aging interventions therefore alter the slope of a measure over time and are detectible statistically as an age-by-compound interaction. An advantage to this slope-based evaluation approach to traditional healthspan metrics is that it enables us to identify and remove intervention hits that are merely general stimulants. Anti-aging compounds may have different effects on lifespan and health if: (1) lifespan and health are separable phenotypes, (2) there are multiple causes of aging, or (3) the intervention acts on a symptom of aging instead of on the underlying cause. A compound’s effects on health and aging can therefore be classified along two axes and into four different categories (Figure 1). The first category (upper right-hand quadrant), represents both lifespan extension and a reduced rate of health decline, resulting in a healthy lifespan extension. The second category (lower right-hand quadrant) represents health preservation at the cost of lifespan. The third category (lower left-hand quadrant) represents general toxicity, with a decrease in lifespan and an increase in the rate of health decline. Interventions that fall into the final category extend lifespan while accelerating health decline (upper left-hand quadrant), which results in a relative extension of the gerospan [18]. Evaluation of data within this conceptual framework enables us to assess a compound’s effect on both lifespan and multiple healthspan parameters as measured across a variety of diverse genetic backgrounds.

To evaluate compound effects in this framework we selected three compounds (NP1, resveratrol, and propyl gallate) previously shown by the CITP to have a positive impact on lifespan in three distinct *C. elegans* test strains (NP1 29% median lifespan increase, resveratrol 24% median lifespan increase, and propyl gallate 22% median lifespan increase; all lifespan increases averaged across the three *C. elegans* strains), although not in *C. briggsae* [34]. NP1, a drug-like chemical, is thought to prolong lifespan by acting as a dietary restriction mimetic [40]. Red wine sirtuin potentiator resveratrol, which has garnered particular interest in the aging field, may extend lifespan via a dietary restriction mechanism different than for NP1 [41]. Propyl gallate was initially tested for lifespan effects in *C. elegans* N2 based on its antioxidant properties [42] and acts through a wholly distinct longevity mechanism. We tested health metrics here using the same chemical lots and frozen strain siblings to the published longevity outcomes [34], with CITP standards for reproducibility across test sites [43].

### Oxidative stress response varies in a compound-, strain-, and age-specific manner

Reactive oxygen species can serve as critical signaling molecules that promote healthy biology, but when ROS production and/or defenses become imbalanced with age, ROS can promote aging [44, 45]. Cells experience oxidative stress due to metabolic activity and environmental stressors. The ability to resist oxidative stress is fundamental to the maintenance of cellular function [46], and therefore oxidative stress resistance is commonly used measure of animal health. Of note, *C. elegans* N2 lifespan has been linked to oxidative stress resistance, with long-lived *C. elegans* insulin pathway mutants demonstrating increased survival under oxidative stress [27]. We sought to assess whether NP1, resveratrol, and propyl gallate, compounds that showed species- and strain-specific effects on lifespan, would exhibit the same pattern of effects on oxidative stress resistance, and if NP1, propyl gallate or resveratrol could confer health effects in strains that did not respond to those compounds with lifespan increases. We tested oxidative stress resistance in the presence of superoxide generator paraquat at two ages: early mid-life (adult day 6 for *C. elegans*, adult day 8 for *C. briggsae*; time points selected based on our previously detailed survival analyses in these genetically diverse backgrounds). We also tested older ages for the *C. briggsae* strains (adult day 16 for *C. briggsae* vs. adult day 12 for *C. elegans*, as *C. briggsae* display a later age of senescent decline [34]) to investigate whether compound effects on oxidative stress resistance were age dependent. Specifically, the different time assays enable us to assess if longevity-promoting compounds ameliorate age-related declines in oxidative stress resistance, rather than merely increasing oxidative stress resistance overall.

**Figure 2:**
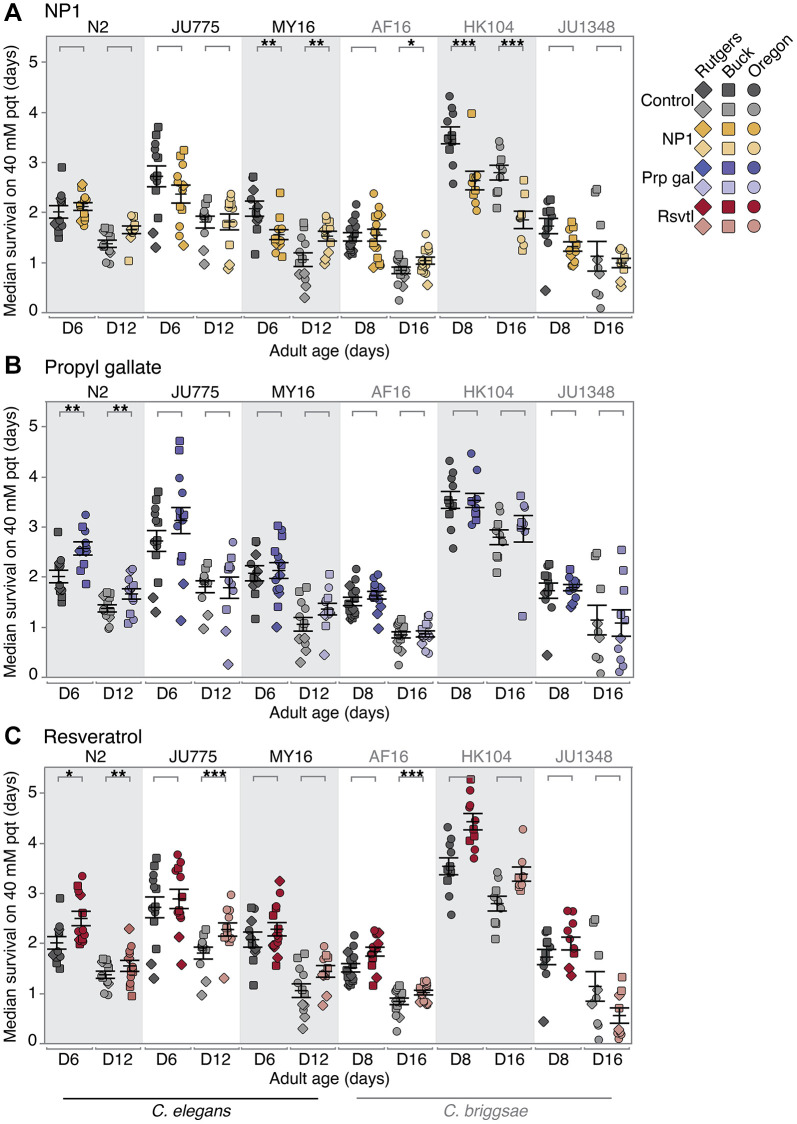
**Compound effects on oxidative stress resistance.** The effect of adult exposure to (**A**) NP1, (**B**) propyl gallate, and (**C**) resveratrol on median survival under oxidative stress conditions, beginning at day 6 and 12 (*C. elegans*), or day 8 and 16 (*C. briggsae*) of adulthood. Three strains were tested from each species: *C. elegans* strains N2, MY16, and JU775 (black text), and *C. briggsae* AF16, ED3092, and HK104 (gray text). Each point represents the median survival on 40 mM paraquat of an individual trial plate (technical replicate), control (vehicle only – gray) or compound treated (color). The bars represent the mean +/− the standard error of the mean. Biological replicates were completed at the three CITP testing sites (square – Buck Institute, circle – Oregon, and diamond – Rutgers). Asterisks represent *p*-values from the CPH model such that ^****^p < 0.0001, ^***^p < 0.001, ^**^p < 0.01, and ^*^p < 0.05.

We found the effects of DR mimetic NP1 on oxidative stress resistance to vary in tested strains across both *elegans* and *briggsae* species (Figure 2A; Supplementary Figure 1). In particular, the strong species-specific effect that we previously reported for lifespan with NP1 treatment [34] was not evident for oxidative stress resistance. More specifically, one strain, *C. briggsae* AF16, showed a small age-related increase in oxidative stress resistance (no significant effect at early mid-life; 15% increase in median survival at late mid-life, *p* = 0.0288). A second strain, *C. elegans* MY16, demonstrated an age-by-compound interaction, with a moderately negative oxidative stress response at early mid-life (25% decrease in median survival, *p* = 0.00135), but a robust positive response at late mid-life age (61% increase in median survival, *p* = 0.00477). *C. briggsae* HK104 had an overall robust decrease in oxidative stress resistance at both ages (33–35% decrease in median survival, *p* < 0.001; we note that this mirrors the decreased lifespan previously seen with HK104 NP1 treatment [34]). The three remaining strains *C. elegans* N2 and JU775, and *C. briggsae* JU1348, showed no significant effect of NP1 treatment on oxidative stress resistance. We conclude that NP1 treatment in genetically diverse backgrounds induces a range of oxidative stress resistance responses. Importantly, for NP1, oxidative stress response does not correlate well with previously reported longevity benefits across strains [34].

Our previously published lifespan results showed that the antioxidant propyl gallate had a weak but positive effect on lifespan in *C. elegans*, and no effect on lifespan in *C. briggsae* [34]. Much like NP1, we found that in most cases propyl gallate did not lead to increased oxidative stress resistance (five of the six strains tested, Figure 2B). Only one strain, *C. elegans* N2, showed an overall increase in oxidative stress resistance, which was more robust in early mid-life (36% increase in median survival at early mid-life, *p* = 0.00128; 24% increase at late mid-life, *p* = 0.00323).

Finally, we tested the effect of resveratrol on oxidative stress resistance, another compound that we showed confers a species-specific lifespan response [34]. Unlike NP1 and propyl gallate, resveratrol had a widespread effect, and increased oxidative stress resistance to statistical significance in three of the six strains tested (Figure 2C). *C. elegans* N2 exhibited an increase in oxidative stress resistance at both ages (27% increase in median survival at early mid-life, *p* = 0.01061; 17% increase at late mid-life, *p* = 0.00688), while *C. elegans* JU775 exhibited increased survival in an age-dependent manner (no significant effect at early mid-life; 13% increase in median survival at late mid-life, *p* = 0.001988). *C. elegans* MY16, along with *C. briggsae* AF16 and HK104 trended towards a general increase in oxidative stress resistance, although not all effects were significant. The remaining strain, *C. briggsae* JU1348, showed no significant change in oxidative stress resistance with resveratrol treatment.

Overall, NP1 and propyl gallate, compounds that CITP previously observed to increase lifespan [34] have little to no effect on the oxidative stress resistance healthspan metric; resveratrol, however, exerted a positive effect on oxidative stress resistance in a species- specific manner.

We conclude that resistance to paraquat toxicity-associated oxidative stress does not correlate strongly with CITP longevity outcomes for select compounds NP1, propyl gallate or resveratrol.

### Thermotolerance varies in a compound-, species-, strain-, and age-specific manner

Thermotolerance has been implicated as a reasonably close correlate of increased longevity [20, 47], with long-lived *C. elegans* mutants being relatively thermotolerant (physiological temperatures for wild-type N2 *C. elegans* range from 15–25°C; thermotolerance is typically described as increased survival after transient exposure to noxious temperatures (above 26°C to at least 36°C)) [48]. To determine the thermotolerance effects of NP1, propyl gallate and resveratrol, we aged animals at 20°C in the presence of the compound and then subsequently measured survival after shifting culture to 32°C on compound-free plates (see Supplementary Figure 2 for Kaplan-Meier survival curves).

**Figure 3:**
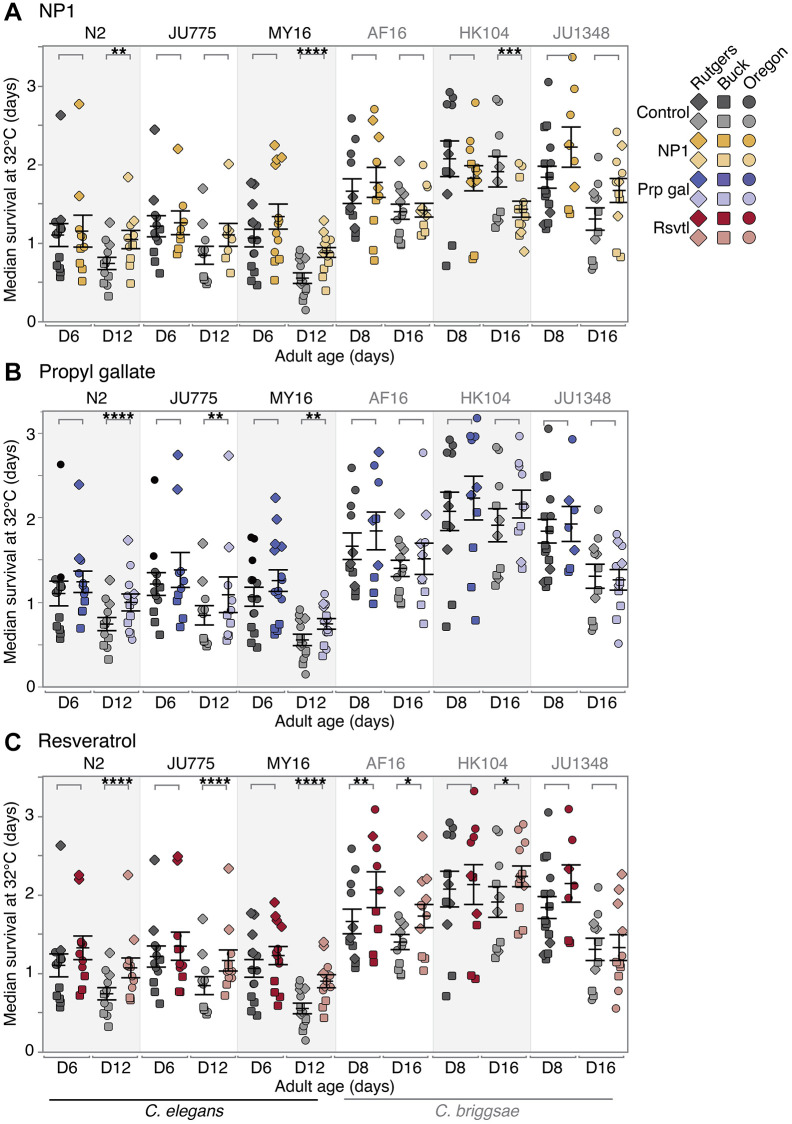
**Compound effects on thermotolerance.** The effect of adult exposure to (**A**) NP1, (**B**) propyl gallate, or (**C**) resveratrol on thermotolerance, specifically median survival at 32°C. Thermotolerance assays were run beginning on day 6 and 12 (*C. elegans*) or day 8 and 16 (*C. briggsae*) of adulthood. Three strains were tested from each species: *C. elegans* strains N2, MY16, and JU775 (black text), and *C. briggsae* AF16, ED3092, and HK104 (gray text). Each point represents the median survival at 32°C of an individual trial plate (technical replicate), either control (vehicle only – gray), or compound treated (color). The bars represent the mean +/− the standard error of the mean. Biological replicates were completed at the three CITP testing sites (square – Buck Institute, circle – Oregon, and diamond – Rutgers). Asterisks represent *p*-values from the CPH model such that ^****^p < 0.0001, ^***^p < 0.001, ^**^p < 0.01, and ^*^p < 0.05.

We found that NP1 ameliorated age-related thermotolerance decline in two of the six *Caenorhabditis* strains tested (Figure 3A), *C. elegans* N2 and MY16 (no significant change at early mid-life; 42–53% increase in median survival at late mid-life; *p* = 0.00611- N2; *p* < 0.0001 – MY16). Conversely, we found that *C. briggsae* HK104 + NP1 exhibited accelerated loss of thermotolerance late in life (no effect at early mid-life; 28% decrease in median survival at late mid-life, *p* < 0.001).

Propyl gallate treatment conferred a moderate positive late life increase in thermotolerance in all three *C. elegans* strains tested (Figure 3B; no significant effect at early mid-life; 9–34% increase in median survival at late mid-life, *p* < 0.0001 – N2, *p* = 0.00138 – JU775, *p* = 0.00238 – MY16). Within this group, the effect size varied in a strain-specific manner. In contrast, thermotolerance in the *C. briggsae* strains tested was not affected by propyl gallate treatment.

Finally, resveratrol caused a significant increase in thermotolerance in both species and five of the six strains (Figure 3C). More precisely, we saw a compound-by-age interaction in four of the six strains tested. These strains exhibited an increase in ability to withstand thermal stress in an age-dependent manner (no effect at early mid-life; 12–51% increase in median survival at late mid-life, *p* < 0.0001 for all *C. elegans* strains, *p* < 0.05 for *C. briggsae* HK104). *C. briggsae* AF16 was positively affected overall by resveratrol treatment, with an increase in survival at both ages tested (34% increase in median survival at early mid-life, *p* = 0.00186; 17% increase at late mid-life, *p* = 0.03046), although the size of the effect decreased with age. The remaining strain, *C. briggsae* JU1348, showed no change in thermotolerance with resveratrol treatment.

Overall, we find the heat resistance associated with lifespan-extending compounds varies with compounds for tests of NP1, propyl gallate, and resveratrol in a genetically diverse test set. NP1 treatment conferred strain- and age-dependent thermotolerance, while propyl gallate elicited generally species- and age-specific responses. We found that resveratrol conferred the most robust and widespread thermotolerance, increasing heat stress survival in nearly every strain, with most strains exhibiting age-dependent effects, and the effect at the younger ages being non-significant but trending towards an increase. Notably, however, thermotolerance outcomes do not correlate with oxidative stress outcomes. We conclude that distinct measures of stress response can be differentially regulated by the compound interventions we tested.

### The effect of compounds on locomotory health is largely species- specific

Previous work has shown that motility decreases in aging *C. elegans* [3, 16, 21, 49]. As locomotory ability is used in human clinical health assessment assays, we investigated whether lifespan-extending compounds would improve *C. elegans* motility, particularly later in life. To measure locomotion features, we used the CeleST platform [49, 50] to acquire eight measures of swimming ability at days 5, 9, and 12 of adulthood, respectively (the selected timepoints showed age-dependent differences in baseline measurements, i.e., are associated with detectable aging between the timepoints); see Supplementary Figure 3 for results with the eight measures of movement; swimming features measured by CeleST are improved in long-lived *C. elegans* mutants [49]. While the eight measures provide a broad range of information on the swimming of each of the strains, it was not clear which measures best capture the decline of locomotion with age across our genetic diversity panel. We therefore combined the information from all eight measures for each strain into a single multivariate composite measure using a linear discriminate analysis to weight and combine the individual measurements (Materials and Methods, Supplementary File, and Online Materials [51] provide an in-depth description of methodology). This approach accounts for interdependency among measures and the unique movement properties of the different strains. We used a strain-specific composite score in our analyses.

**Figure 4:**
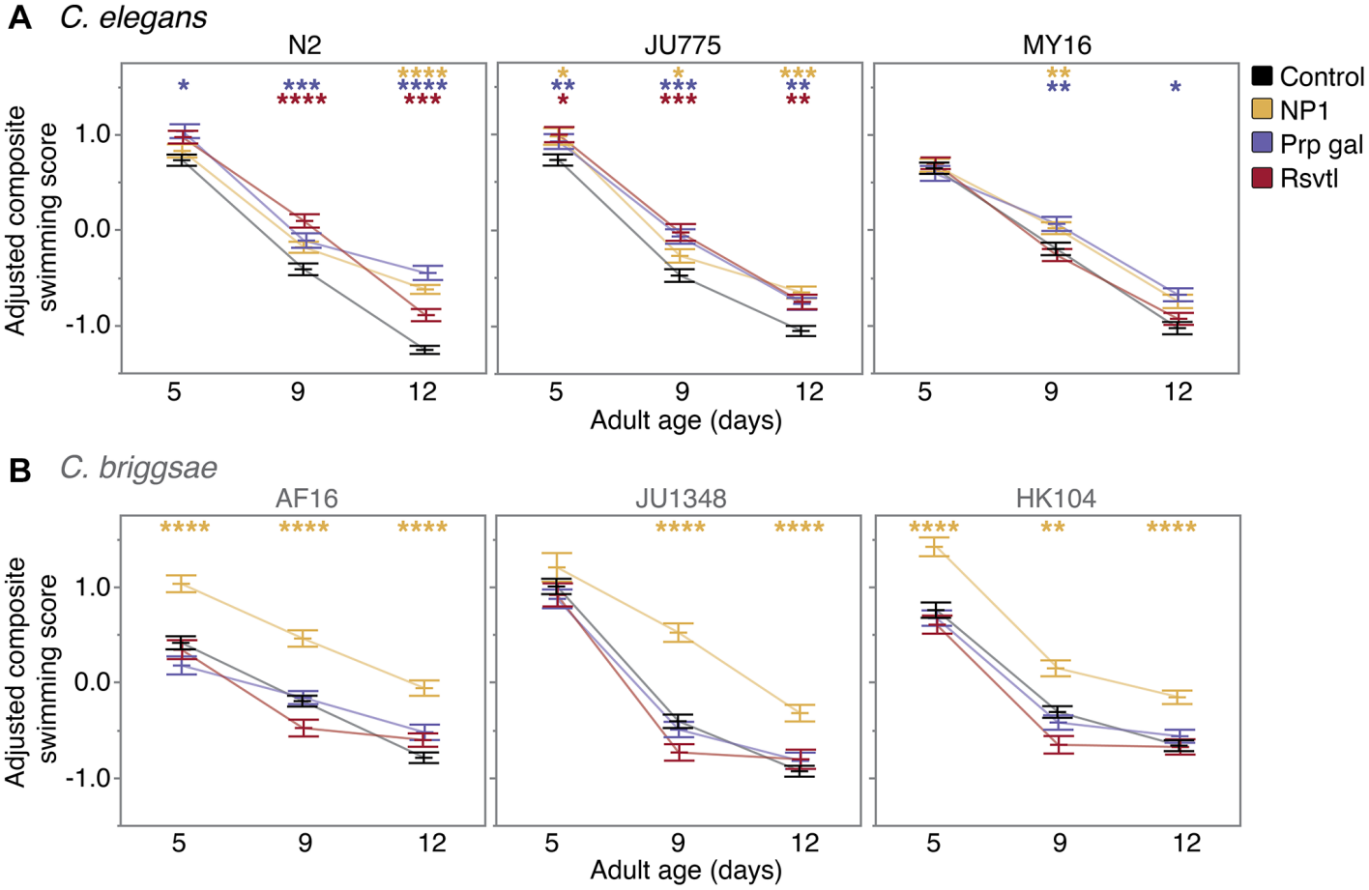
**Compound effects on CeleST composite swim scores.** The effect of adult exposure to NP1, propyl gallate, or resveratrol on overall swimming ability with age in (**A**) three *C. elegans* strains (N2, JU775, MY16), and (**B**) three *C. briggsae* strains (AF16, JU1348, HK104). Swimming assays were run on days 5, 9, and 12 of adulthood. Bars represent the mean +/− the standard error of the mean. Adjusted swimming score values were normalized to the strain mean value. Two biological replicates were completed at each of the three CITP testing sites. Asterisks represent *p*-values from the linear mixed model such that ^****^p < 0.0001, ^***^p < 0.001, ^**^p < 0.01, and ^*^p < 0.05.

We found that NP1 had the most robust and widespread effect on swimming ability, improving locomotion in all six strains tested (Figure 4). In *C. elegans*, the effect of NP1 was age-dependent, with NP1 treatment slowing the rate of decline in swimming ability in all strains. We observed the largest effect in *C. elegans* N2 (17% increase in mean swimming score at day 12, *p* < 0.0001), while *C. elegans* JU775 and MY16 exhibited small but significant improvements (2–5% increase with age, *p* < 0.05 – JU775; 3% increase at adult day 9, *p* = 0.0085 – MY16). Interestingly, the effect of NP1 on swimming ability in *C. briggsae* was strikingly robust. JU1348 showed a reduction in age-related locomotory decline (12–16% increase in mean swimming score at adult days 9 and 12, *p* < 0.0001), while both AF16 and HK104 had an overall increase in swimming ability at all ages (9–14% increase in mean swimming score, *p* < 0.0001 for all ages with the exception of HK104 day 9, where *p* = 0.0028). Although NP1 conferred an overall increase in the locomotory ability of HK104, the effect was greatest in young animals, meaning that NP1 increased the relative rate of decline in strain HK104.

We found that propyl gallate treatment improved swimming ability in a species-specific manner, similar to the propyl gallate effect on lifespan (Figure 4). All three *C. elegans* strains showed an age-related improvement in swimming ability, with the effect size increasing at the older ages tested. Propyl gallate improved locomotory ability in N2 by 5% (*p* = 0.0105) at adult day 5, 7% (*p* = 0.0005) at day 9, and 21% (*p* < 0.0001) at day 12. This trend was also observed in both JU775 and MY16 to a lesser extent (4–6% maximum increase in mean composite swimming score during adult days 9 and 12; *p ≤* 0.0003 – JU775, *p* < 0.05 – MY16), indicating that propyl gallate reduces the rate of age-related locomotory decline in *C. elegans*. In contrast, propyl gallate treatment had no effect on swimming ability in any *C. briggsae* strain tested.

We found the effect of resveratrol on locomotory ability was also species-specific (Figure 4). Specifically, resveratrol treatment improved swimming ability with age in *C. elegans* JU775 and N2 (5–11% increase in mean composite swimming score at day 9, 4–10% increase at day 12; *p* ≤ 0.0001 – N2, *p* ≤ 0.0015 – JU775), slowing the age-related decline in locomotion in both strains. This effect, however, is dependent on genetic background, as swimming ability in *C. elegans* MY16 was not affected by resveratrol. Likewise, resveratrol treatment had no effect on swimming in any *C. briggsae* strain.

Overall, we found that compounds that extend lifespan in particular strains also improve mobility when administered to those same strains. It is noteworthy, however, that we also found that NP1, which did not have a lifespan effect in the *C. briggsae* genetic background, could nonetheless enhance adult swimming ability. Thus, NP1 extends locomotory health more broadly than it enhances longevity in a genetically diverse test set.

### Thermotolerance, but not oxidative stress or swimming ability, correlates with lifespan

Do health measurements correlate with lifespan, especially in response to a pharmacological intervention? Because our health assays were terminal (for throughput considerations and need to minimize manipulation stress, we did not recover animals used in the swimming assays for use in parallel lifespan studies), we do not have health and lifespan measurements for the same individuals. However, we were able to correlate each health measure score on a combined strain, compound, and age basis (Supplementary Figure 4) and found that thermotolerance was the most predictive of median lifespan, particularly at days 12 (*C. elegans*) and 16 (*C. briggsae*) of adulthood (R^2^ = 0.58 at early mid-life, R^2^ = 0.83 at late mid-life). In contrast, neither oxidative stress resistance nor swimming ability correlated well with median lifespan, with swimming ability on the days assayed being a particularly poor predictor of the effect of a compound on longevity (R^2^ = 0.08, 0.097, 0.048, for days 5, 9, and 12 of adulthood, respectively). Our data suggest that despite experimental variability in thermotolerance outcomes, thermal resistance might be the best proxy for screening for longevity promoting drugs, an idea that remains to be tested more extensively.

### Swimming ability and oxidative stress resistance, but not thermotolerance, are highly reproducible across labs

We observed some variability within our healthspan datasets and statistically evaluated the sources of this variation (Table 1). For oxidative stress resistance, ~34% of the total variance is attributed to genetic background, ~7.8% of total variance is attributed to lab-specific effects, and ~9.8% of the total variance is a result of variation within labs. This distribution of variation is similar to the variation we observed in our previously published lifespan datasets [34, 38]. The sources of variation within the swimming dataset are weighted towards individual variation, with ~79.2% of total variance attributable to differences in individual swimming performance. Variability at the genetic level only accounts for 5.7% of the variance, with nearly half of that (2.4%) attributable to species differences. Furthermore, among-lab differences only contributed to ~5.8% of the total variation, and within lab variance contributed to ~9.3% of the total variation. Both oxidative stress resistance and swimming are thus reproducible across labs.

**Table 1:** Comparison of reproducibility of health measurements from oxidative stress, thermotolerance, and CeleST assays within and between labs.

**Source of variation**	**Oxidative stress**	**Thermo**	**CeleST**
**Genetic variation**	**34.0**	**24.4**	**5.7**
Among species	0.0	0.6	2.4
Among strains w/in species	29.2	3.7	0.9
Species × age	0.1	1.4	0.1
Species × compound	0.0	0.0	0.8
Species × age × compound	0.0	15.3	0.2
Strain × age	0.0	1.0	0.7
Strain × compound	3.8	1.9	0.1
Strain × age × compound	0.9	0.6	0.6
**Reproducibility among labs**	**7.8**	**28.5**	**5.8**
Among labs	3.5	13.8	3.3
Lab × species	0.0	7.5	0.6
Lab × strain	3.6	0.8	1.1
Lab × age	0.5	4.8	0.6
Lab × compound	0.0	0.7	0.0
Lab × age × compound	0.1	0.9	0.2
**Reproducibility within labs**	**9.8**	**15.8**	**9.3**
Among trials	4.1	6.8	
Among scanners w/in trials	0.0	3.6	
Among plates w/in scanners	5.8	5.4	
Among experimenters			0.8
Among trials w/in exptrs			2.0
Among videos w/in trials			6.5
**Individual variation**	**48.4**	**31.3**	**79.2**
**Total**	**100**	**100**	**100**
Total number of observations	20,467	22,213	15,117

We found thermotolerance outcomes to be variable in comparison to longevity, locomotion and oxidative stress measures. For thermotolerance, ~24.4% of the total variation is attributed to genetic background, while we find ~28.5% of variation is attributable to among lab differences, and ~15.8% to variation within labs. Variability may reflect difficult-to-control environmental factors such as localized/fluctuating differences in incubator temperature (or temperature distribution), or humidity variation. Regardless of root cause, however, data reveal that even against the backdrop of variability in thermotolerance, the thermotolerance measure may serve as a plausible tool for assessing the chances that a given intervention might exert longevity benefit.

## DISCUSSION

The ultimate goal of exploiting model organisms to screen for anti-aging interventions is to identify treatments that might translate to healthy lifespan extension in humans (Figure 1). Few people are interested in living to be 120 years old if longevity is associated with continuing health declines seen in most 90-year-olds, but most would be happy to live to 100 with a health state similar to 60-year-olds. Despite widespread application of the longevity screening approach, it remains somewhat unclear as to whether the goal of translating model organism research to healthy human aging is achievable, in part because the relationship between healthspan and lifespan varies based on how/when health is measured, analyzed, and interpreted [17, 18, 52, 53]. Using varying measures of health, some compound interventions exhibit independent effects on health and lifespan [31–33]. That a disconnect between phenotypes of health and longevity is surprising likely reflects typically unspoken assumptions regarding the mode of action of interventions, and the underlying causation of the age-dependent phenotypes of health and lifespan.

For example, compound interventions that treat the root cause(s) of aging would be expected to confer broad age-dependent health benefits, as well as reduced mortality. Yet, it is possible to treat symptoms of aging instead of underlying causes. In those cases, if the symptom is only related to mortality or to a particular health measure, we would expect separation between lifespan and health effects. It is therefore generally beneficial when characterizing anti-aging interventions to ask: are there broad health benefits indicative of a fundamentally slowed aging process? And is that aging process influenced by genetic background?

### Genetic background can profoundly affect the efficacy of a tested intervention to promote survival or health

Although many screens for longevity extension in *C. elegans* have been published [54–57], genetic background effects remain an unexplored variable in these screens as nematode studies have been largely confined to the canonical *C. elegans* N2 lab strain. The CITP seeks to address the issue of genetic background variability in compound screening by testing a panel of *Caenorhabditis* strains that represent evolutionary divergence similar to that found between mice and humans [35–37]. As we have previously published, genetic background is important when assessing the effects of pharmacological interventions on lifespan [34]. Here we selected three anti-aging compounds (NP1, propyl gallate, and resveratrol) for which we had previously conducted comprehensive studies of longevity [34] and we characterized their impact on two measures of physiological resilience, oxidative stress resistance (Figure 2) and thermotolerance (Figure 3), and on swimming ability (Figure 4), a primarily neuromuscular phenotype thought to reflect general health and vigor.

**Figure 5:**
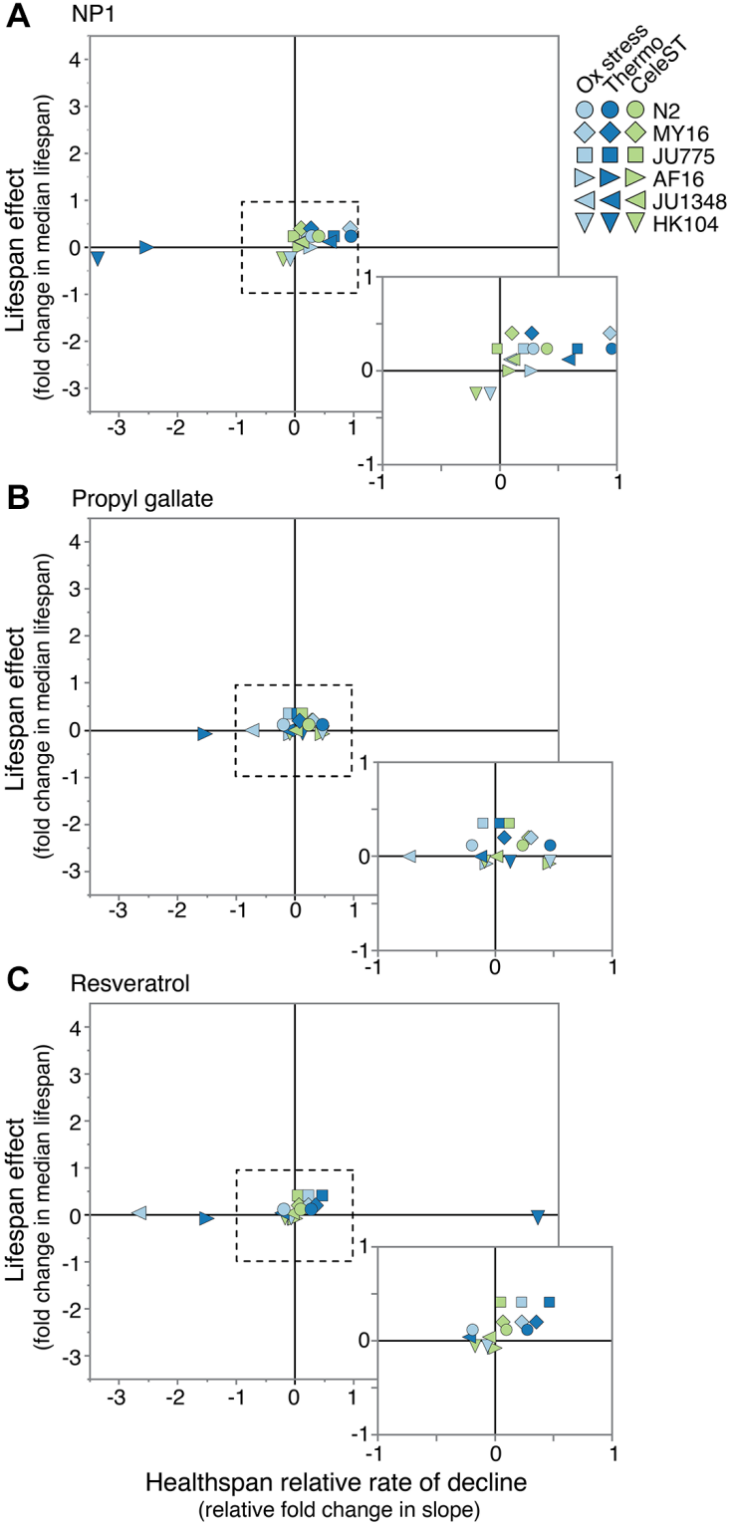
**Relative compound effects on health vs. lifespan.** Comparing the effect of (**A**) NP1, (**B**) propyl gallate, and (**C**) resveratrol on manual lifespan versus healthspan measures in two *Caenorhabditis* species (lifespan data from reference [34]). The lifespan effect is the fold change in median lifespan for a strain compared to its untreated control. For health, the relative rate of decline for each strain and compound is compared to the rate of decline for the control. Positive numbers would reflect either lifespan extension or slowed decline in the health measure, while negative numbers would reflect shortened lifespan and accelerated decline in the health measure. Each point represents a strain and health measure combination. Dotted line surrounds expanded box.

To determine if these three compounds induced lifespan extension by affecting a root cause in the aging process, we determined whether they conferred broad health benefits. We observed that if a compound extended lifespan in a particular strain, that compound typically slowed the rate of decline across the healthspan measures tested, with some exceptions (Figure 5). This raises an interesting question: if a compound modulates a core aging process, then why are the observed compound effects dependent on the genetic background? This may result from background differences in permeability, compound turnover, and/or pathway tuning that can vary with genetic background, or may suggest that the underlying cause(s) of aging varies across genetic backgrounds, or may indicate that the compounds are affecting symptoms of aging instead of causes of aging. This separation in phenotypes suggests that interventions that do not exhibit a benefit in one age-dependent measure may show benefits when assayed using an alternative measure. We therefore sought to determine if compound interventions promote health benefits in genetic backgrounds that do not exhibit lifespan extension. Interestingly, pharmacological interventions in the strains that saw no lifespan change after compound treatment had varying and sometimes robust effects on the rate of health decline, both positively and negatively. In this way, although health effects can vary, we observe that healthspan can generally be uncoupled from lifespan, and that anti-aging effects of compounds could be seen on health regardless of lifespan effects.

### Thermotolerance as a proxy for longevity outcomes?

Given the relationship among different health measures and lifespan, we sought to determine which health measures are most reproducible and informative for targeted screens for anti-aging treatments. In the case of the three health measures that we evaluated, thermotolerance was the only one for which compound intervention effects correlated well with lifespan effects across genetic backgrounds (Supplementary Figure 4). Increased thermotolerance corresponds to increased longevity in many studies [20, 58–60]. The fact that lifespan correlates significantly with thermotolerance across compounds and genetic backgrounds suggests that the ability to maintain cellular homeostasis is a key factor in not just maintaining health, but also crucial in preventing death. Thermal stress causes unfolding of cellular proteins, and the induction of stress response pathways that induce organelle-specific molecular chaperone production [61, 62]. We therefore suggest the correlation between lifespan and thermotolerance as being likely attributed to compound administration promoting protein homeostasis, which when addressed could result in lifespan extension and increased thermotolerance.

Despite the relationship of thermotolerance to lifespan, thermotolerance may only be used sparingly in future CITP compound screening for three reasons: (1) given the correlation between thermotolerance and lifespan, there may be little additional benefit for using thermotolerance assays given that positive hits will likely be identified by lifespan studies alone, (2) although in principle thermotolerance provides a faster result, the approach used here actually ended up being more labor intensive than standard longevity assays (see Methods), and (3) most importantly, among our three assays, thermotolerance was the least reproducible across labs (Table 1).

### Oxidative stress resistance and swimming locomotion reflect aging processes that are uncorrelated with lifespan

In contrast to thermotolerance, oxidative stress resistance, the other physiological resilience measure we used in these studies, does not correlate well with lifespan (Supplementary Figure 4). Although oxidative damage has been proposed as a cause for aging [63–65], this may not be surprising for two reasons. First, ROS signaling plays a crucial role in multiple cellular processes [45] and certainly the positive and negative aspects of ROS signaling play into the outcome equation [44]. Indeed, it is known that antioxidant treatment does not guarantee increased lifespan, indicating that oxidative stress may not be a primary driver of mortality [31–33]. Secondly, “oxidative stress” is an umbrella term that covers a wide range of phenomena, with sub-cellular localization of the oxidative stress and differing reactive oxide species resulting in differing levels of cellular stress or benefit. In this study we only tested a severe oxidative stressor, paraquat, and our results may have been different had we used a lower concentration of paraquat, a different oxidative stressor, or a more physiological deleterious ROS source.

Similar to oxidative stress, we found that swimming ability, a measure of neuromuscular function [66], does not correlate well with median lifespan across the different genetic backgrounds tested (Supplementary Figure 4). While oxidative stress resistance and swimming ability do not predict ultimate lifespan, these measures remain of particular interest when exploring the effect of pharmacological interventions on aging. Of note, the measures of oxidative stress and swimming prowess showed high reproducibility among labs (Table 1), and their separability from lifespan can facilitate the identification of interventions that treat important symptoms of aging that may not be found by screening lifespan alone.

Here we documented that representative pharmacological interventions in a genetically diverse *Caenorhabditis* test set result in a complex pattern of health effects, with genetic background and age both being important for overall outcome. Assay selection and design is clearly a factor in evaluation of intervention impact. Compounds can improve health measures in the absence of strong longevity outcome. Our results underscore that genetic background can be a crucial determinant in the evaluation of potential anti-aging, pro-resilience compounds. Although the findings presented here are limited to three compounds relevant to human interventions, our findings suggest that a personally tailored intervention that accounts for age, current health state, and genetic background may ultimately be necessary for optimal efficacy in the clinic.

## MATERIALS AND METHODS

A detailed set of standard operating procedures is available online [67]. Experimental details in brief are as follows:

### Strains

Following standard CITP protocol, the following natural isolates were obtained from the *Caenorhabditis* Genetics Center (CGC) at University of Minnesota: *C. elegans* N2, MY16, JU775; *C. briggsae* AF16, HK104, JU1348. The N2 strain used was N2-PD1073, which is a clonal line derived from the N2 strain VC2010 used to generate a new N2 reference (VC2010-1.0) genome [68] that has been adopted by the CITP as a lab adapted control [38]. Animals were maintained at 20°C on 60 mm plates with NGM and *E. coli* OP50-1 and synchronized by timed egg-lays (for full SOP see ref. [67]). Animals were transferred to 35 mm NGM plates containing 51 μM FUdR and compound intervention (or the solvent DMSO in control plates) on the first, second and fifth day of adulthood, then once weekly when applicable, until healthspan measurements were initiated.

### Interventions

The following compounds were selected to study impacts on health based on our previous findings for nematode longevity [34, 38]: NP1 (ChemBridge), propyl gallate (Sigma-Aldrich), and resveratrol (Cambridge Chemical). The same in-plate concentrations previously tested for lifespan effects from each compound were used here: 50, 200, and 100 μM, respectively. Animals were exposed to compound interventions only for the duration of adulthood up until health measurements were performed. Lifespan data for *C. elegans* and *C. briggsae* strains are from Lucanic et al. (2017) [34]; healthspan studies were conducted on identical strain stocks between 2017 and 2022.

### Selection of ages for health assays

To measure health in aging adults we selected time points that (1) showed age-dependent differences in the baseline measurements (*e.g.*, detectable aging between the timepoints), and (2) were justifiable based on the known physiological and demographic changes in normally aging adults. For example, *C. elegans* and *C. briggsae* hermaphrodites are self-fertile, with a normal reproductive period in the absence of males lasting for the first five to eight days of adulthood [69]. We therefore used the end of the period of self-reproduction to establish an “early-mid-life” timepoint for health assays. We also selected a “late-mid-life” timepoint to correspond to approximately the 95th survivorship centile [70] to minimize selection biases (see Supplementary Figure 5).

### Heat stress

To measure the impact of each compound on organismal tolerance of heat stress, we implemented the following augmented Lifespan Machine (LM) protocol [38, 71]: 70 animals each placed on 50 mm tight-lidded petri plates with modified NGM and *E. coli* OP50-1 at 32°C humidity for a duration of four days. For each of two biological replicates (independent trials), we created two technical replicates (single plate) per strain and condition (age and compound or control) per lab. *C. elegans* were tested at adult days 6 and 12 while *C. briggsae* were tested at adult days 8 and 16. A full protocol is available online [67].

### Oxidative stress

To measure the impact of each compound on organismal resilience to exogenous oxidative stress, we implemented the following ALM protocol [38, 71]: 70 animals were placed onto each 50 mm tight-lidded petri plate with modified NGM containing 40 mM paraquat (or methyl viologen dichloride, from Sigma-Aldrich), 51 μM FUdR, and *E. coli* OP50-1. The duration of the paraquat-exposure assays was dependent on the starting age of the animals due to the increased rate of mortality with age. Specifically, *C. elegans* on day 6 of adulthood and *C. briggsae* on day 8 of adulthood were assayed for 16 days, while *C. elegans* at 12 days of adulthood and *C. briggsae* at 16 days of adulthood were assayed for only 7 days. For each of two biological replicates, we created two technical replicates per strain and condition (age and compound or control) per lab. A full protocol is available online [67].

### Heat and oxidative stress statistical analysis

Statistical analyses for survival were conducted as previously published [34, 38]. In brief, a mixed-model approach within each strain was used in which compound and age were treated as fixed effects, and laboratory site, experiment date, lifespan machine, and technical replicate were considered random effects. Effects of compounds on healthspan were tested via the compound-by-age interaction, with the compound or control specific change with age being defined as the “rate of decline” of that healthspan measure. We analyzed survival using both a general linear model with the lme4 (v1.1.23) [72] package and a mixed model Cox proportional hazard (CPH) model with the coxme (v2.2.16) package [73]. Each compound was tested as a planned comparison against its appropriate control using the multcomp (v1.4.13) package [74]. Analyses were conducted in the R statistical language. All relevant data, R-scripts, and output files are available online [75].

### *C. elegans* swim test

To measure the impact of lifespan-enhancing compounds on the age-associated decline in agility or neuromuscular function, we implemented the *C. elegans* Swim Test (CeleST [49]). Briefly, animals were exposed to compound intervention during adulthood as described above until CeleST measurements were collected at adult ages day 5, 9, and 12. For two biological replicates at each of the three CITP sites, 40 animals were tested per condition (age and compound or control) per strain. For full experimental protocols see our online protocol [67]. The CeleST software was used to measure eight different parameters (Wave initiation rate, Body wave number, Asymmetry, Stretch, Curling, Travel speed, Brush stroke, and Activity index) [49, 50]. To facilitate comparisons between strains and compound treatments we generated a single composite swimming score.

### CeleST composite score

CeleST provides video-based analysis of eight separate features of locomotion, from bending rate to travel speed^50^. However, we do not necessarily have an a priori expectation as to how each variable might change with age, or how strains may differ in age-dependent changes among the eight measures. To maximize the differences between ages across all the measurements, we used linear discriminant analysis (LDA), with the eight original measurements in each record serving as the predictor variables and age-dependent decline being the primary discriminator (see Supplementary File) to reduce the number of dimensions from eight to one. Projecting the eight predictor variables onto a single axis creates the first linear discriminant function. This first linear discriminant function minimizes within-age variance, maximizes between-age variance, and maximizes the separability of the means of the ages [76]. While it cannot capture all the information provided by each original measurement, it captures as much as possible. Because it maximizes the differences between group levels (ages in our case), linear discriminant analysis is often used to predict group membership after a training dataset is used. To avoid confounding strain specific differences in swimming, our LDA was performed on the untreated control data independently for each strain to generate first linear discriminant functions for each strain. This function provides the composite movement score that best captures the decline in movement with age. We then used the coefficients for each of the original eight predictor variables in the strain specific first linear discriminant functions as strain specific weightings of the eight measures to generate composite scores that maximized the ability to separate the animals in the control set by age. Those strain specific weightings were then used to generate a composite score of treated animals to analyze for compound and age effects (see Supplementary File for in depth methodology). While the main text presents an analysis of swimming using the composite score, analyses of the individual CeleST measurements are available in the Supplementary Materials (Supplementary Figure 3). All relevant R-scripts and raw output are available online [51, 67]. To analyze changes in swimming behavior with age, the composite score was used as the dependent in mixed effects general linear models built for each strain in R using the lme4 package (v1.1.27.1) [72], with compound and age as fixed effects and laboratory site, research technician, experiment date, and video as nested random effects. Significant age by compound interactions, as determined using the R car package (v3.0.11) [77], were used to determine the effect of the compound on the rate of decline of swimming with age.

## Supplementary Materials

Supplementary File

Supplementary Figures
