# Supplementary material for: The coupling between healthspan and lifespan in Caenorhabditis depends on complex interactions between compound intervention and genetic background
Source: Aging (Albany NY). 2024 Apr 12;16(7):5829–55. doi: 10.18632/aging.205743 (PMC11042945; doi:10.18632/aging.205743)
Supplement: Supplementary Figures [file aging-16-205743-s002.pdf]

SUPPLEMENTARY FIGURES

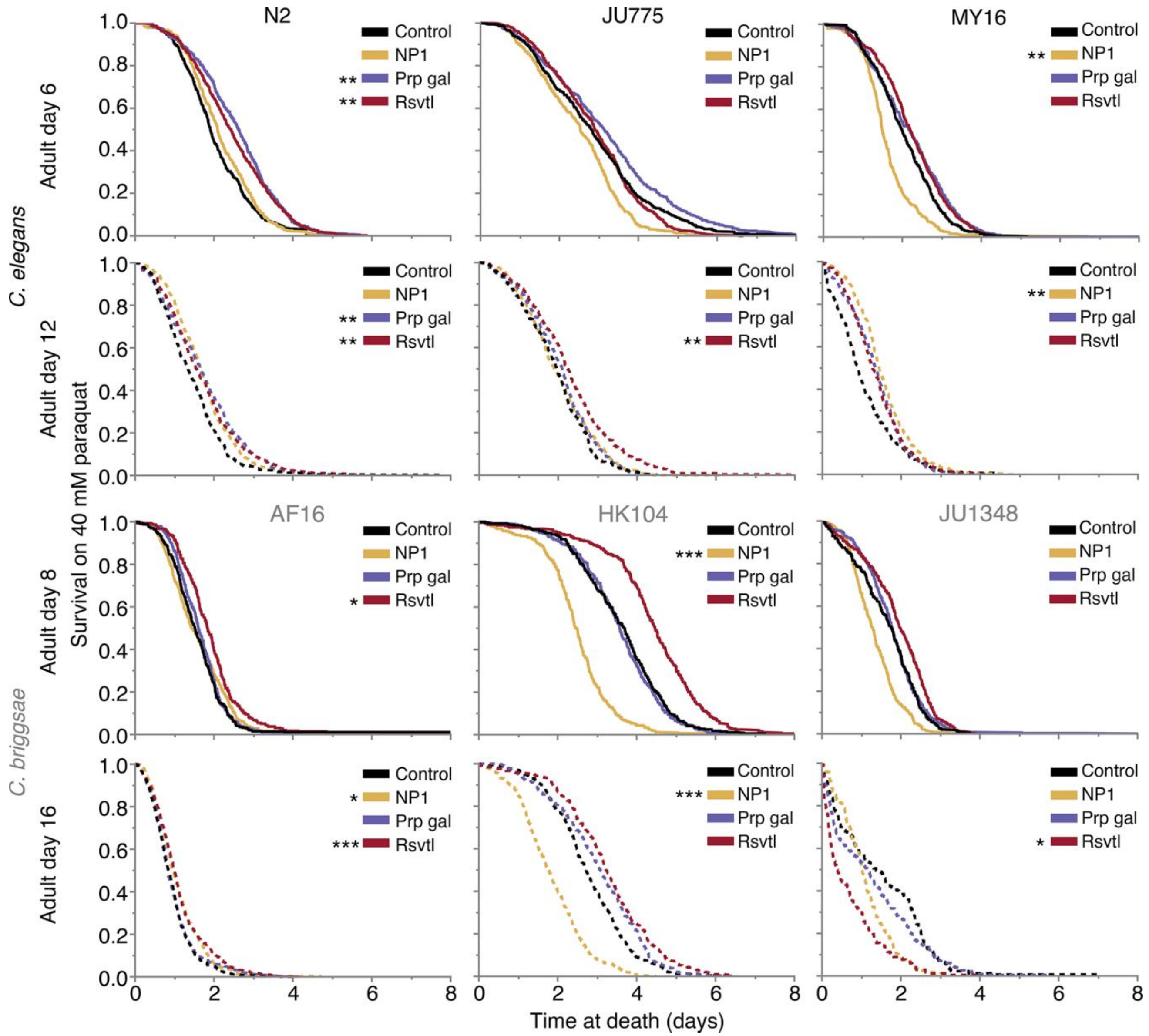

Supplementary Figure 1. Kaplan-Meier curves showing the effect of adult exposure to NP1, propyl gallate, or resveratrol on survival under oxidative stress conditions (40 mM paraquat) beginning at early and late mid-life (adult days 6 and 12 for *C. elegans*, days 8 and 16 for *C. briggsae*). Solid lines indicate early mid-life, dashed indicate late mid-life. Each curve represents multiple biological and technical replicates conducted at each of the three CITP testing sites. Asterisks represent *p*-values from the CPH model such that $^{****}p < 0.0001$, $^{***}p < 0.001$, $^{**}p < 0.01$, and $^{*}p < 0.05$.

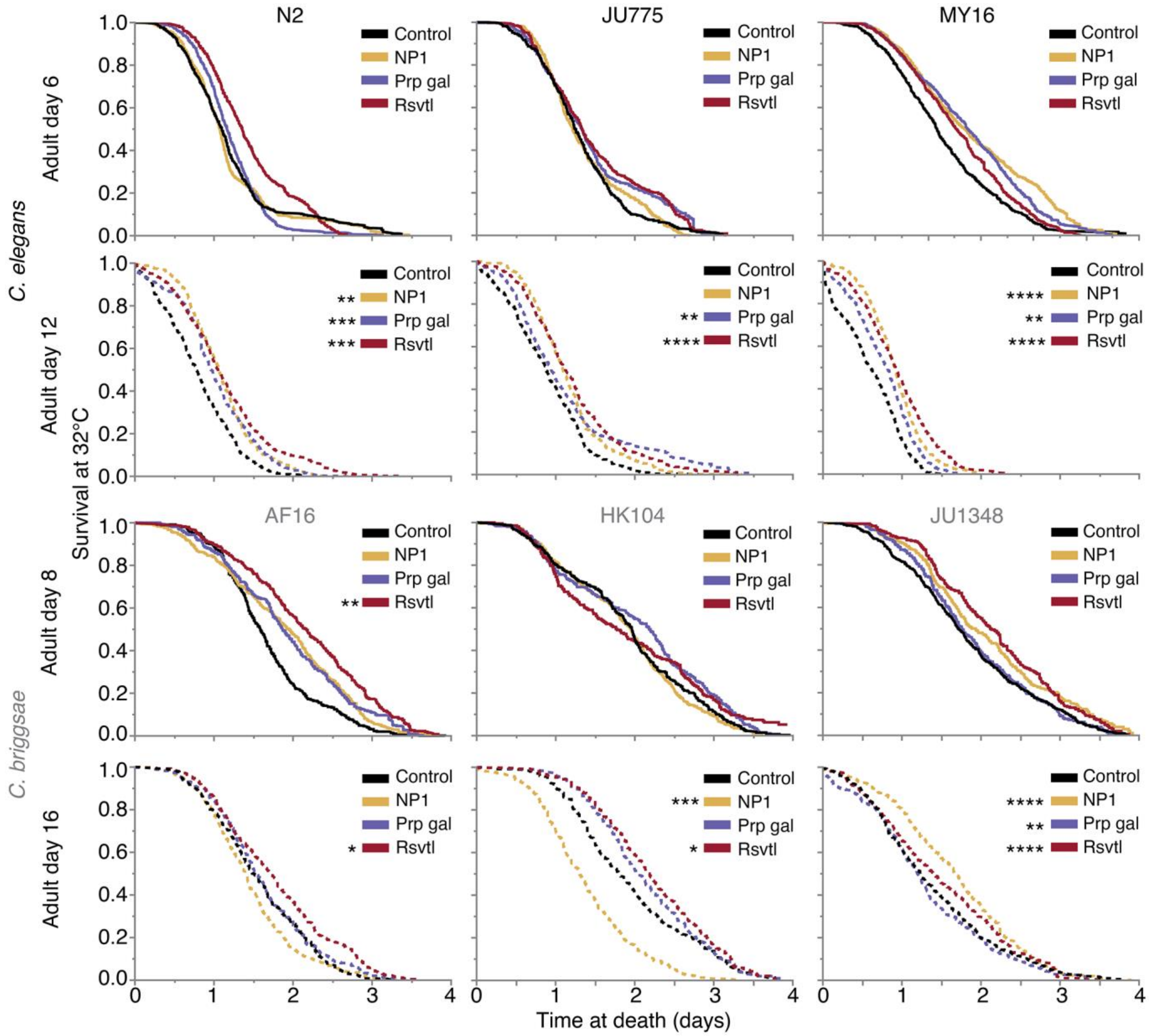

Supplementary Figure 2. Kaplan-Meier curves showing the effect of adult exposure to NP1, propyl gallate, or resveratrol on thermotolerance at 32°C beginning on day 6 and 12 (*C. elegans*) or day 8 and 16 (*C. briggsae*) of adulthood. Solid lines indicate early mid-life, dashed indicate late mid-life. Each curve represents multiple biological and technical replicates conducted at each of the three CITP testing sites. Asterisks represent *p*-values from the CPH model such that $^{****}p < 0.0001$, $^{***}p < 0.001$, $^{**}p < 0.01$, and $^{*}p < 0.05$.

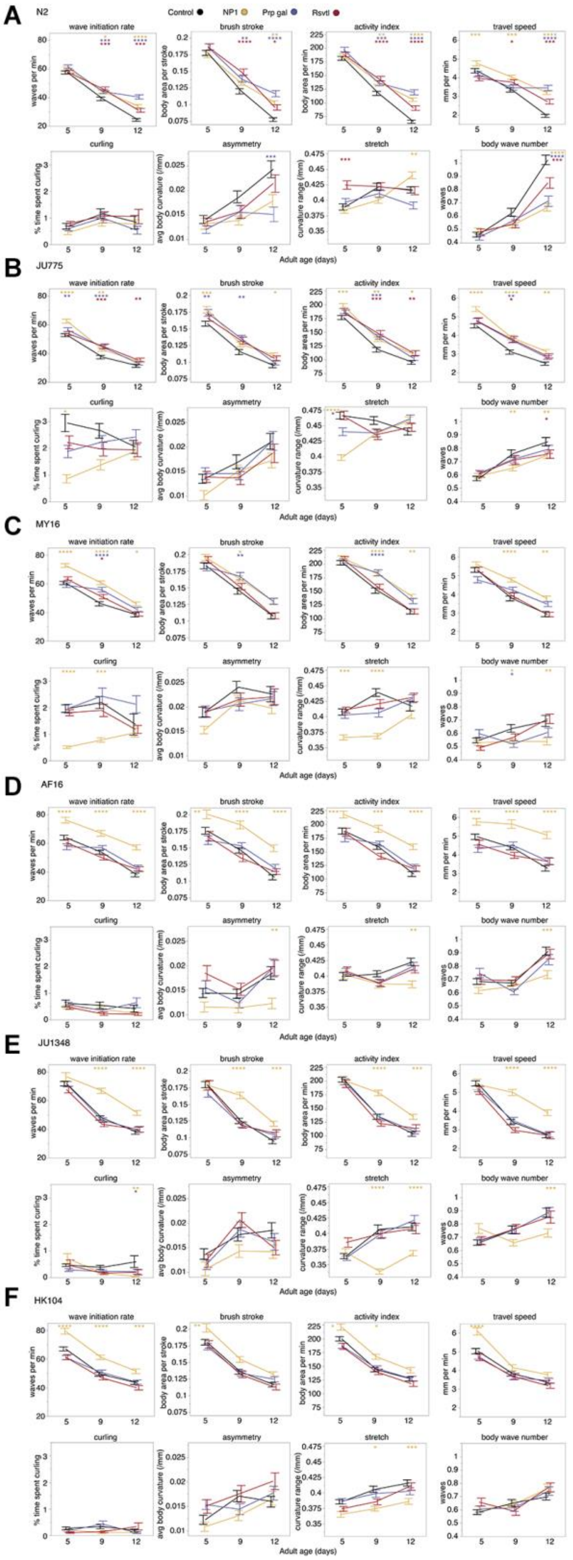

Supplementary Figure 3. The effect of adult exposure to NP1, propyl gallate, or resveratrol on eight measures of swimming ability with age. Data is shown for three *C. elegans* strains: (**A**) N2, (**B**) JU775, and (**C**) MY16, and three *C. briggsae* strains: (**D**) AF16, (**E**) JU1348, and (**F**) HK104. Swimming assays were run at early mid-life, mid-life, and late mid-life (days 5, 9, and 12 of adulthood, respectively). The line represents the mean of an individual trial, bars represent the mean +/− the standard error of the mean, and the colors correspond to the compound treatment (black-control, yellow-NP1, purple-propyl gallate, red-resveratrol). Two biological replicates were completed at each of the three CITP testing sites. Asterisks represent *p*-values from the linear mixed model such that $^{****}p < 0.0001$, $^{***}p < 0.001$, $^{**}p < 0.01$, and $^{*}p < 0.05$.

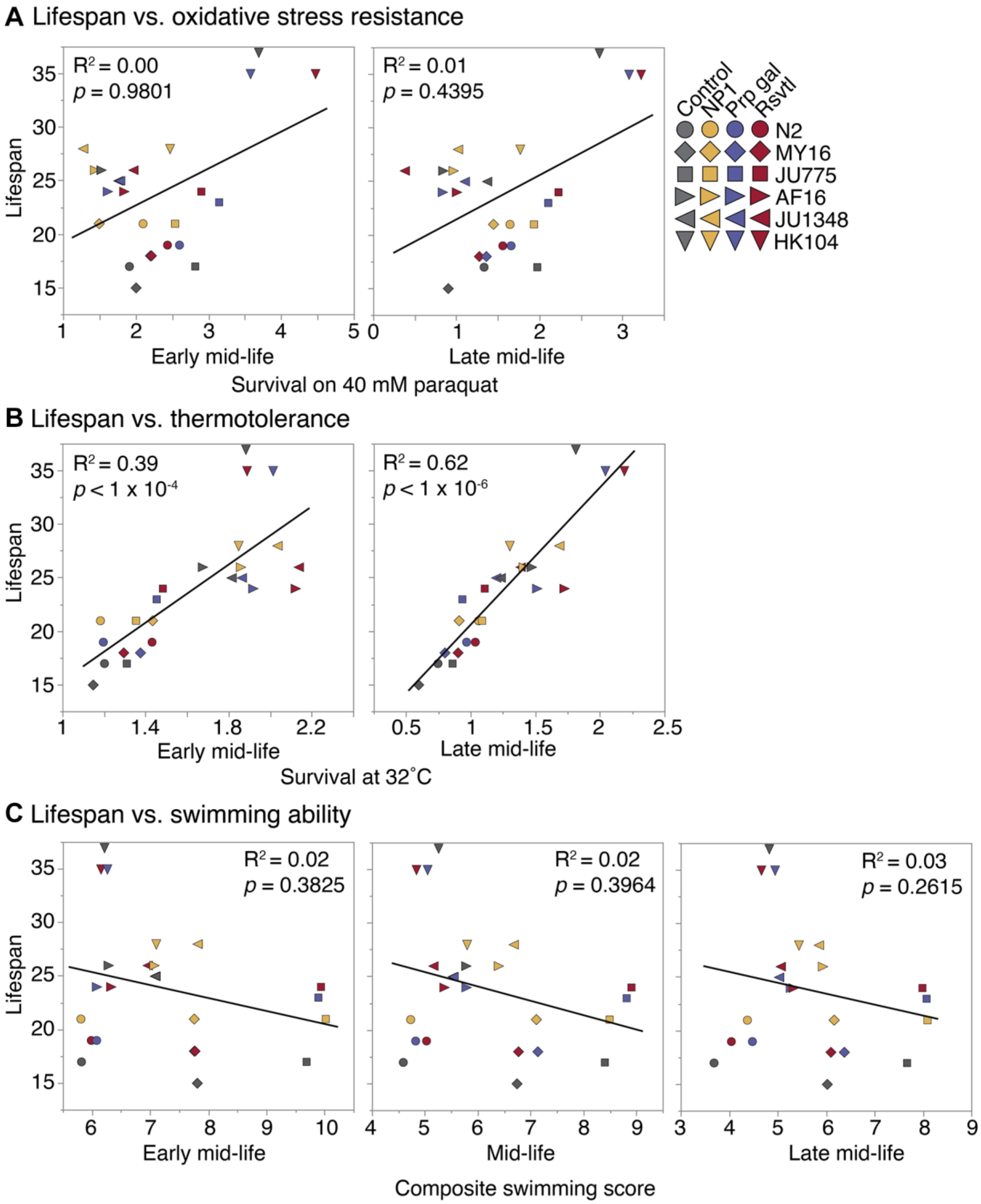

Supplementary Figure 4. Correlation between lifespan and age-specific health measures after pharmaceutical intervention. Health was measured at early and late mid-life for all assays, with an additional measurement at mid-life for swimming ability. Median values were used for composite swimming ability, while Kaplan-Meier medians were used for lifespan, (**A**) oxidative stress resistance, and (**B**) thermotolerance, while median values were used for (**C**) composite swimming ability. Kendall's tau correlation coefficients were calculated using data from three *C. elegans* strains (N2, MY16, and JU775) and three *C. briggsae* strains (AF16, JU1348, and HK104) across multiple compound interventions (DMSO controls, NP1, propyl gallate and resveratrol). The only significant correlation with lifespan was for thermotolerance, with R2 = 0.39 for early mid-life, and R2= 0.62 at late mid-life (p < 0.0001).

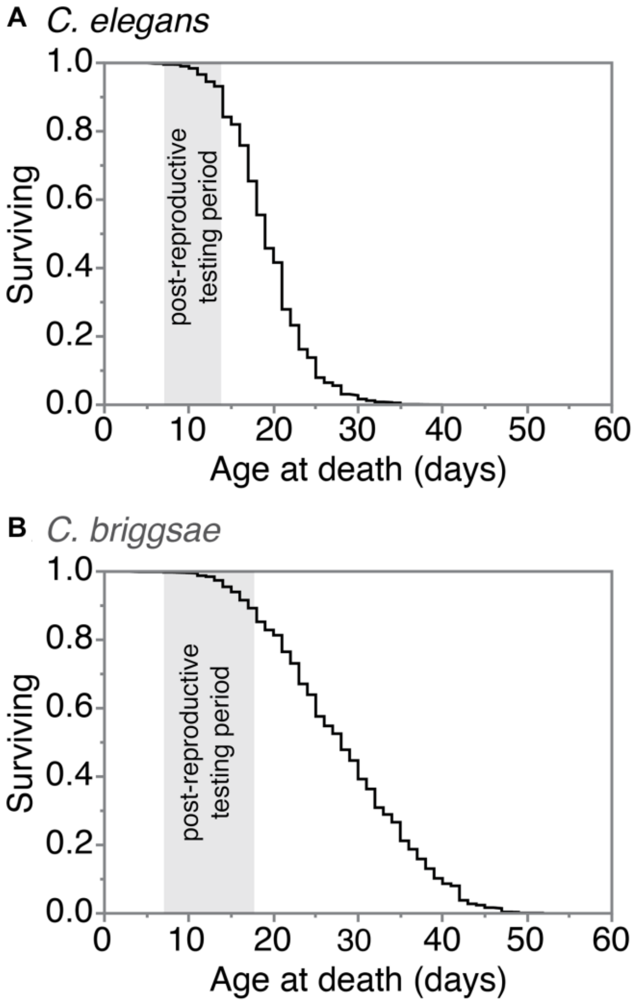

Supplementary Figure 5. Kaplan-Meier lifespan curves showing overall species lifespan curves for (**A**) *C. elegans*, and (**B**) *C. briggsae*. Each curve consists of our previously published baseline survival data [34] from the three strains tested within a given species: N2, JU775, and MY16 for *C. elegans*, and AF16, JU1348, and HK104 for *C. briggsae*. Ages tested for health metrics were chosen based on the end of reproduction up until the (approximate) 5% quantile of survival under baseline conditions.
